# Insights from the analysis of draft genome sequence of *Crocus sativus* L.

**DOI:** 10.6026/97320630018001

**Published:** 2022-01-31

**Authors:** Sheetal Ambardar, Jyoti Vakhlu, Ramanathan Sowdhamini

**Affiliations:** 1 National Center for Biological Sciences, Bellary Road, Bengaluru, India; 2 School of Biotechnology, University of Jammu, J&K, India; 3 Institute of Bioinformatics and Applied Biotechnology, Bengaluru 560100, India

**Keywords:** Crocus sativus, de-novo genome assembly, apocarotene biosynthesis pathway, MYB TFs, SSR markers, Orthology analysis

## Abstract

Saffron (Crocus sativus L.) is the low yielding plant of medicinal and economic importance. Therefore, it is of interest to report the draft genome sequence of C. sativus. The draft genome of C. sativus has been assembled using Illumina sequencing and is 3.01 Gb long covering 84.24% of genome. C. sativus genome annotation identified 53,546 functional genes (including 5726 transcription factors), 862,275 repeats and 964,231 SSR markers. The genes involved in the apocarotenoids biosynthesis pathway (crocin, crocetin, picrocrocin, and safranal) were found in the draft genome analysis.

## Background:

Plant genomics, with the increasing number of whole genome sequences available, has unlocked the genetic treasures that would be impossible in absense of the genome sequence. Though second and third generation sequencing technologies, coupled with ever advancing bioinformatic tools/pipelines, have made the sequencing of complex and huge genomes economical and easy, but till date there are only approximately 1886 plant genome sequences available in databanks (NCBI: https://www.ncbi.nlm.nih.gov/assembly). Some of the recently sequenced and assembled plant genomes are rice [1], maize [2], asparagus [3], wheat [4] and tea [5] etc., however the genome of the plants belonging to Crocus genus or Iridaceae family, have not been reported so far. Saffron (C. sativus) referred as 'Golden Condiment' is world's most expensive spice costing about 70,000 INR/pound, with medicinal properties and cosmetic uses [6]. More than 150 volatile and aroma-yielding compounds contribute to the flavor, color, and aroma of the saffron spice, wherein the main chemical constituents in the stigma of saffron are crocin, crocetin, picrocrocin, and safranal [7]. C. sativus is an autumn-flowering perennial sterile triploid plant (2n = 24) with, ∼3.5 Gb haploid genome [8, 9]. Being sterile, it fails to produce viable seeds and reproduces vegetatively by underground corms and is reported to lack genetic variation. Various molecular markers (RAPD, ISSR, AFLP, SSR microsatellites) and epigenetic approaches have suggested the existence of limited genetic variability [10-13]. To discover authentic genetic markers, mining genes for secondary metabolites and improvement of breeding, sequencing of its genome was the only alternative. In addition, it's ancestry is also controversial that could be also settled, if its complete genome sequence is available [14,15]. Hybrid sequencing approaches, comprising of second and third generation sequencing technologies, have facilitated sequencing of complex genomes economically. Illumina sequencing technology is preferred in combination of other sequencing technologies for first sequencing attempt, as it generates good sequencing data for better genome coverage and has low error rate as compared to third generation sequencing technologies [16]. Therefore, it is of interest to document data to gain insights from the preliminary analysis of draft genome sequence of Crocus sativus L. It should be noted that a draft version of this article has been made open access at the Biorxiv repository [17].

## Materials and methods:

C. sativus corms were collected from Kishtwar, J&K (33.3116° N, 75.7662° E) in 2019. Corms were grown in the pots for period of three months and leaves were harvested for genome size estimation. Genome size of the plant was estimated by flow cytometric (Hare and Johnston 2011) and k-mer based method using Jelly Fish [18]. Genomic DNA was extracted from corm tissue using CTAB method [19] and quality and quantity was accessed using Qubit (Invitrogen) and agarose gel electrophoresis. 3 microgram DNA was used to construct WGS DNA libraries with 550bp and 800bp insert sizes using NEB next Ultra DNA Library Preparation Kit according to the Illumina's protocol. Quality of the libraries was evaluated using Tapestation (Agilent 4200) and Qubit HS DNA Assay Kit (Invitrogen) and sequenced on HiSeqX platform (150-bp paired-end (PE) reads) to generate 321 Gb data (∼92X coverage). Quality of raw reads was evaluated using FastQC tool [20] and low quality bases (>q30) and sequencing adapters were removed using trimmomatic software [21]. De-novo genome assembly was performed using Soapdenovo2 [22] and MaSuRCA [23]. Soapdenovo2 assembly was executed using different kmers (73 kmer predicted by KmerGenei along with 69, 71 kmers) [24]. The statistics of soapdenovo2 assemblies were compared to select the better assembly that was designated as Cs_Assembly_1. MaSuRCA assembly was done using the raw reads and was designated as Cs_Assembly_2. The quality of assemblies was accessed using BUSCO against Viridiplantae lineage from OrthoDB database [25]. Subsequently, raw illumina reads were mapped back to Cs_Assembly_2 using Bowtie2 [26] and previously published transcriptome data [27, 28] was mapped to Cs_Assembly_2 using BWA [29].

Repetitive regions in Cs_Assembly_2 was identified using Repeatmasker and GenomeScope v2 [30,31] and SSR markers were identified using MISA [32]. Cs_Assembly_2 was further analysed for gene prediction using the MAKER [33] wherein C. sativus transcriptome data was used as EST evidence [28], Viridiplantae database (UNIPROT) as protein evidence, maize as Augustus gene prediction model and Oryza sativa as snap hmm. Predicted proteins were further annotated using BLASTp against NR (NCBI) and viridiplanteae (UNIPROT) database with modified parameters (E-value-1e-3, sequence identity >40% and query coverage >70%). Annotated proteins were analysed for GO annotations against biological processes, cellular component and metabolic processes using WEGO [34]. Transcription factors (TFs) proteins were identified against PlantTFDB [35] using BLASTp with the modified parameters (E-value-1e−3, sequence identity >30%, query coverage >70%). Orthologous genes were comparedwith Asparagus officinalis, Phalaenopsis equestris, Apostatia shenzhenica of the same plant order along with Oryza sativa (Rice) using Orthovenn2 [36]. The proteins sequences of all the plants were downloaded from Phytozome database [37]. Various metabolic pathways in C. sativus genome were analysed using KAAS webserver [38].

## Data availability:

Whole genome sequencing raw reads and draft genome of Crocus sativus has been submitted to NCBI SRA under bioproject PRJNA734464 and PRJNA739096 respectively. All the processed data including draft genome, annotated proteins, and supplementary tables can be accessed at CAPS_NCBS server [39].

## Results & Discussion:

**Figure 1:**
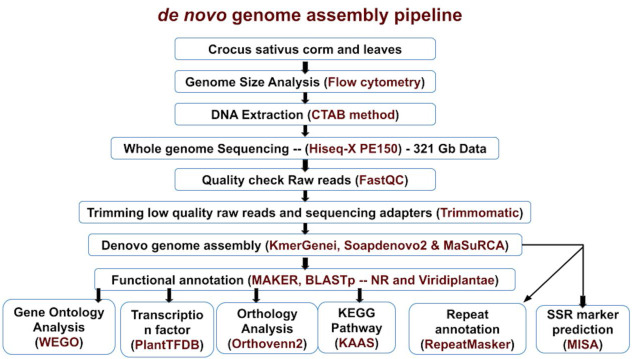
Schematic of de-novo genome assembly and annotation pipeline. Black colour text represents the analytical processes and Red colour text represents the software/instrument used to perform the processes.

Crocus sativus genome is the first draft genome sequence of the plant belonging to the Iridaceae family. Genome size of C. sativus was estimated to be 3.5 Gb (3,578,575,507 bases), using flow cytometry and kmer method. Genome size estimated was comparable to earlier reports, wherein it was estimated to be 3.44 Gb using flow cytometry being grown in Italy, Spain and Israel [8,9]. On the basis of size of the genome, 321 Gb WGS data of C. sativus was generated, with an overall coverage of ∼92X using Illumina sequencing (Supplementary Table 1 - see PDF). De-novo genome assembly and annotation of C. sativus was performed using the bioinformatics pipeline represented in Figure 1. De-novo genome assembly using Soapdenovo2 with kmer 71 was comparatively better than other two kmers (69 and 73) and was designated as Cs_Assembly_1 with N50 value of 1596 and 77.9% genome coverage (Table 1 - see PDF). De-novo genome assembly with MaSuRCA was designated as Cs_Assembly_2 with N50 value of 1860 and 84.24% genome coverage. Cs_Assembly_2 was found comparatively better than Cs_Assembly_1 as the assembly statistics, such as N50, largest scaffold, genome coverage and BUSCO completeness were higher in Cs_Assembly_2 than Cs_Assembly_1. (Table 1 - see PDF). Further, ∼87.28% of raw reads mapped back to Cs_Assembly_2, thereby indicating that most of data has been utilized for genome assembly. In addition, two previously published transcriptome data sets [28,29] were mapped to the Cs_Assembly_2 and mapping percentage of 99.92% and 92.02% were observed against Cs_Assembly_2 (Supplementary Table 2 - see PDF). High mapping percentage represented the presence of most of the reported exons/CDS in the Cs_Assembly_2 even though the genome assembly was fragmented with less N50 value. Polygonum cuspidatum genome was de-novo assembled using Soapdenovo2 with Illumina reads and generated an assembly of 2.56 Gb, with N50 value of 3215 and 98.5% genome coverage [40]. Similarly, the genome of Linum usitatissimum, flax plant was de-novo assembled using Illumina reads having N50 scaffold of 694 Kb with 81% of genome coverage [41]. Genome coverage of C. sativus was comparatively more than flax genome but less than Polygonum cuspidatum genome using same sequencing technologies. Total repeats length in C. sativus genome (Cs_Assembly_2) was 1,460,908,750 bp (40.8%) as predicted by Genome Scope version 2. A total of 862,275 repeats were identified in Cs_Assembly_2 wherein simple repeat (48.41%) and LTR (30.34%) were the most abundant in the genome. Specifically, Copia & Gypsy were the most abundant LTR repeats (Supplementary Table 3 - see PDF). A total of 9,64,231 SSR markers were identified in Cs_Assembly_2 wherein monomeric SSR repeats (4,86,140 (50.4%)) were more abundant as compared to dinucleotide (2,94,819 (30.5%)) and trinucleotide repeats (1,46,991 (15.2%)) with "A", "TA","TTG" most abundant SSRs in each groups. The abundance of Tetranucleotide (15,375 (1.59%)), pentanucleotide (8,596 (0.9%)) and hexanucleotides (12,310 (1.27%)) repeats each was less than 2% of total SSRs with "AAAT", "TATAT" and "TAACCC" most abundant in respective SSRs (Supplementary Table 4 - see PDF). SSR markers are reported to be multi-allelic, relatively abundant, widely dispersed across the genome and have been used in genetic diversity analysis, parentage assessment, species identification and mapping genetic linkage [42]. These markers can be further evaluated for their application in C. sativus. Earlier studies on C. sativus transcriptome have reported the presence of 16,721 SSRs [28] and 79,028 SSRs [43] using transcriptome analysis, but higher number of SSR (964,231) were discovered in the present study based on genome sequence.

**Figure 2:**
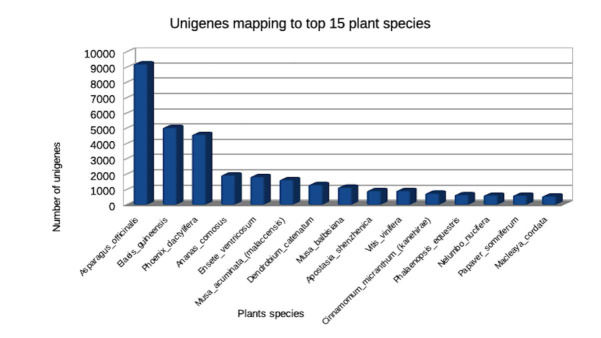
Crocus sativus unigenes mapping to top 15 plant species wherein most of the proteins annotated against Asparagus officinalis.

In total 254,038 proteins were predicted from Cs_Assembly_2 using MAKER pipeline. A total of 52,435 and 52,545 proteins were annotated based on BLASTp against NR and viridiplanteae database respectively (Supplementary Table 5 - see PDF). BUSCO analysis revealed the presence of 75.7% of the plant conserved genes/orthologues in the C. sativus genome. Out of total proteins, 51% (26796) were annotated to 8 top-hit plant species (Figure 2). Maximum number of proteins was annotated against Asparagus officinalis (9213) indicating C. sativus to be phylogenetically closer to Asparagus officinalis, as both the plants belong to same plant order Asparagales (Figure 2). 85% of total proteins (43,649) were associated with gene ontology (GO) ids and classified into biological processes (BP: 22,092 proteins) abundant in cellular and metabolic processes, cellular components (CC: 24,399 proteins) mostly localised in cell and organelle parts and molecular functions (MF: 34,442 proteins) most abundant in catalytic and transporter activities (Supplementary Table 6 - see PDF). Out of the total annotated proteins, 5726 unique C. sativus proteins were identified as transcription factors (TFs) belonging to 57 TFs families. MYB & MYB related family proteins (11.86%), being more abundant TFs, followed by bHLH, C2H2, NAC, FAR1, C3H, ERF, bZIP, WRKY and B3 were the top 10 abundant transcription factors family proteins (Supplementary Figure 1, Supplementary Table 7 - see PDF). TFs like MYB & MYB related, bHLH, WRKY are reported to regulate secondary metabolite (apocarotenoid) biosynthesis in C. sativus [28]. Earlier reports on C. sativus transcriptome has identified less number of TFs (3819, 2601), whereas the most abundant TFs family remains same [27,28].

**Figure 3:**
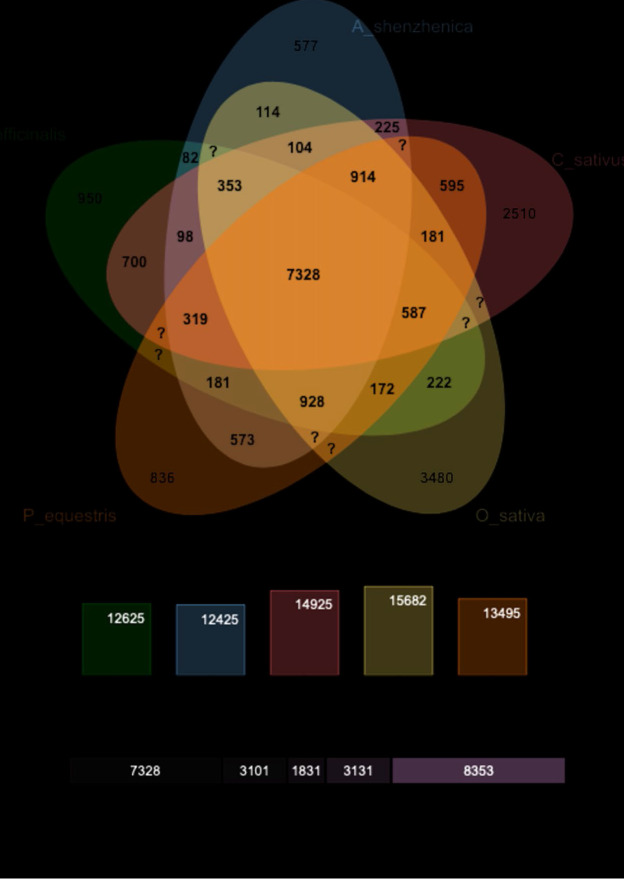
Orthology analysis of Crocus sativus with neighboring plants from same order (Asparagus officinalis, Phalaenopsis equestris, Apostatia shenzhenica) along with Oryza sativa representing 7328 proteins clusters to be conserved in all the five plant species, whereas 2510 proteins cluster were unique to C. sativus only.

C. sativus annotated proteins (52,545) was compared with 3 monocots plants of same order, whose genome and annotations were available in Phytozome database [37], namely Asparagus officinalis, Phalaenopsis equestris, Apostatia shenzhenica along with a model monocot plant Oryza sativa (Rice) using Orthovenn2 (Figure 3). A total of 23,744 proteins cluster were found in all the plants wherein 21,606 were orthologous clusters that were atleast common in two species and 2138 were single copy gene clusters wherein each cluster have only one gene from each plant species. Conservation of 7328 proteins clusters, comprising of 51,803 proteins, was observed among the five species (C. sativus: 10,001 proteins, A. officinalis: 9552, P. equestris: 9012, A. shenzhenica: 8570 and O. sativa: 14,668) (Supplementary Figure 2 - see PDF). The conserved proteins clusters were found to be associated with biological processes (BP-23,010 proteins), cellular component (CC-582 proteins) and molecular functions (MF-957 proteins) and were enriched in defence response, RNA modification, DNA integration, regulation of transcription, rRNA processing and protein phosphorylation (Supplementary Table 8 - see PDF). However, 2510 protein clusters (7914 proteins) were unique to Crocus sativus only, out of which 1636 clusters (4595 proteins) were associated with slimmed GO terms (BP: 5201, CC: 63, MF:303 proteins) associated with nucleic acid binding, transferase, hydrolase, oxidoreductase activity and protein and DNA binding activity (Supplemenatary Table 8 - see PDF). As per orthology analysis also, C. sativus was found phylogenetically closer to A. officinalis as more protein clusters were orthologous between Crocus sativus and Asparagus officinalis than to other plants compared in the study (Supplementary Figure 3 - see PDF).A total of 10,912 C. sativus proteins were mapped to 395 KEGG pathways of monocots. Various pathways like carbohydrate metabolism, energy metabolism, lipid metabolism, nucleotide metabolism, amino acid metabolism, glycan metabolism, metabolism of cofactors and vitamins along with biosynthesis of terpenoids, polyketides and other secondary metabolites were found complete wherein all the genes involved in pathway were present in draft assembly. We further investigated the presence of genes involved in the synthesis of apocarotenoids namely crocins, picrocrocin, and safranal that are produced in the stigma of C. sativus. These apocarotenoids impart red color, bitter taste, and pungent aroma to stigma of saffron and have various medicinal properties [7]. The molecular basis of apocarotenoid biosynthesis in C. sativus has been well studied using transcriptomics studies [27, 28]. In the present study, the genes encoding the enzymes involved in carotene biosynthesis pathway, regulating the apocarotenoids synthesis, were present in the C. sativus genome (Supplementary Figure 4 - see PDF). This is the first de-novo draft genome sequence of Crocus sativus that needs to be complemented with the long read sequencing technology (PacBio) to fill in the gaps in the present genome to generate a complete genome sequence. However, this draft genome sequence, in addition to revealing previous unknown genomic information on saffron, will be used as a reference genome for future genome sequencing attempts in saffron.

## Conclusion:

It is of interest to establish a de-novo reference genome of Crocus sativus for the first time. De-novo assembly of Crocus sativus has been constructed using only Illumina short read, thus, has large number of scaffolds and assembly gaps thereby indicating that our assembly should be referred to as a draft genome sequence. Nevertheless, this study represents the first attempt to assemble the Crocus sativus genome, providing a valuable resource for the community to facilitate future research.
